# Seizure development in the acute intrahippocampal epileptic focus

**DOI:** 10.1038/s41598-018-19675-6

**Published:** 2018-01-23

**Authors:** Lin Li, Kseniia Kriukova, Jerome Engel, Anatol Bragin

**Affiliations:** 1 Department of Neurology, David Geffen School of Medicine at University of California Los Angeles, 710 Westwood Plaza, Los Angeles, CA 90095 USA; 2 Department of Neurobiology, David Geffen School of Medicine at University of California Los Angeles, 710 Westwood Plaza, Los Angeles, CA 90095 USA; 3 Department of Psychiatry and Biobehavioral Sciences, David Geffen School of Medicine at University of California Los Angeles, 710 Westwood Plaza, Los Angeles, CA 90095 USA; 4 Brain Research Institute, David Geffen School of Medicine at University of California Los Angeles, 710 Westwood Plaza, Los Angeles, CA 90095 USA; 5 I.M. Sechenov First Moscow State Medical University, Moscow, Russia

## Abstract

Currently, an epileptic seizure is considered to involve a temporary network that exists for a finite period of time. Formation of this network evolves through spread of epileptiform activity from a seizure onset zone (SOZ). Propagation of seizures evoked by kainic acid injection in hippocampus to different brain areas was analyzed at macro- and micro-intervals. The mean latency of seizure occurrence in different brain areas varied between 0.5 sec and 85 sec (mean 14.9 ± 14.5 (SD)), and it increased after each consecutive seizure in areas located contralateral to the area of injection, but not in the ipsilateral sites. We have shown that only 41% of epileptic individual events in target brain areas were driven by epileptic events generated in the SOZ once the seizure began. Fifty-nine percent of epileptiform events in target areas occurred one millisecond before or after events in the SOZ. These data illustrate that during seizure maintenance, only some individual epileptiform events in areas outside of SOZ could be consistently triggered by the SOZ; and the majority must be triggered by a driver located outside the SOZ or brain areas involved in ictal activity could be coupled to each other via an unknown mechanism such as stochastic resonance.

## Introduction

One of the key questions in epilepsy research is: “How does brain activity synchronize during seizures?” Understanding mechanisms of seizure propagation and maintenance can provide insights into prevention, and more effective treatment, by targeting specific pathways participating in seizure spread. Our traditional understanding of seizure spread is informed by delineation of morphological pathways between brain areas^1–6^. Some seizures quickly spread from the area of onset to numerous target areas. For example, absence seizures originate in neocortex and spread across the brain quickly within several milliseconds^7,8^, which may indicate that they spread via existing morphological pathways. Other seizures propagate from the area of detection to other brain areas within several seconds and tens of seconds^9–12^, indicating the existence of different mechanisms of propagation. One uncertainty in the electrophysiological approach to detecting the location of onset of spontaneous seizures is that the decision concerning the site of seizure onset is based on the first appearance of epileptiform discharges in the subset of recording electrodes within an electrode montage, which covers less than 1% of brain volume. There is always the possibility that, in reality, the seizure began somewhere else. For the same reason, measurements of speed of propagation may be misleading.

In this study, we provide results of analysis of propagation of epileptiform events generated in an acute epileptic focus created by intrahippocampal injection of kainic acid (KA). KA activates kainate glutamate receptors located both on principal cells and interneurons, thereby increasing activity in both types of neurons^13,14^, and simulates seizures that occur due to hyperactivity of both principal cells and interneurons. We specifically focused on the analysis of the process of recruitment of brain areas outside the injection area, considered to be the seizure onset zone (SOZ). In addition to visual estimation of propagation of epileptiform events from the area of occurrence at “macro” intervals, we analyzed the relationship between the SOZ and its potential targets at “micro” intervals, once the seizure began. This analysis was based on ***event by event*** measurements of the temporal relationships between the SOZ and its potential targets. The null hypothesis for this study is that epileptiform activity from the SOZ engages certain target brain areas into the ictal activity along morphological pathways via synaptic and non-synaptic mechanisms, and that the latency of epileptiform events in target brain areas depends on the physical distance between these areas and SOZ.

## Results

### LFP epileptiform patterns in the injection area

**Figure 1:**
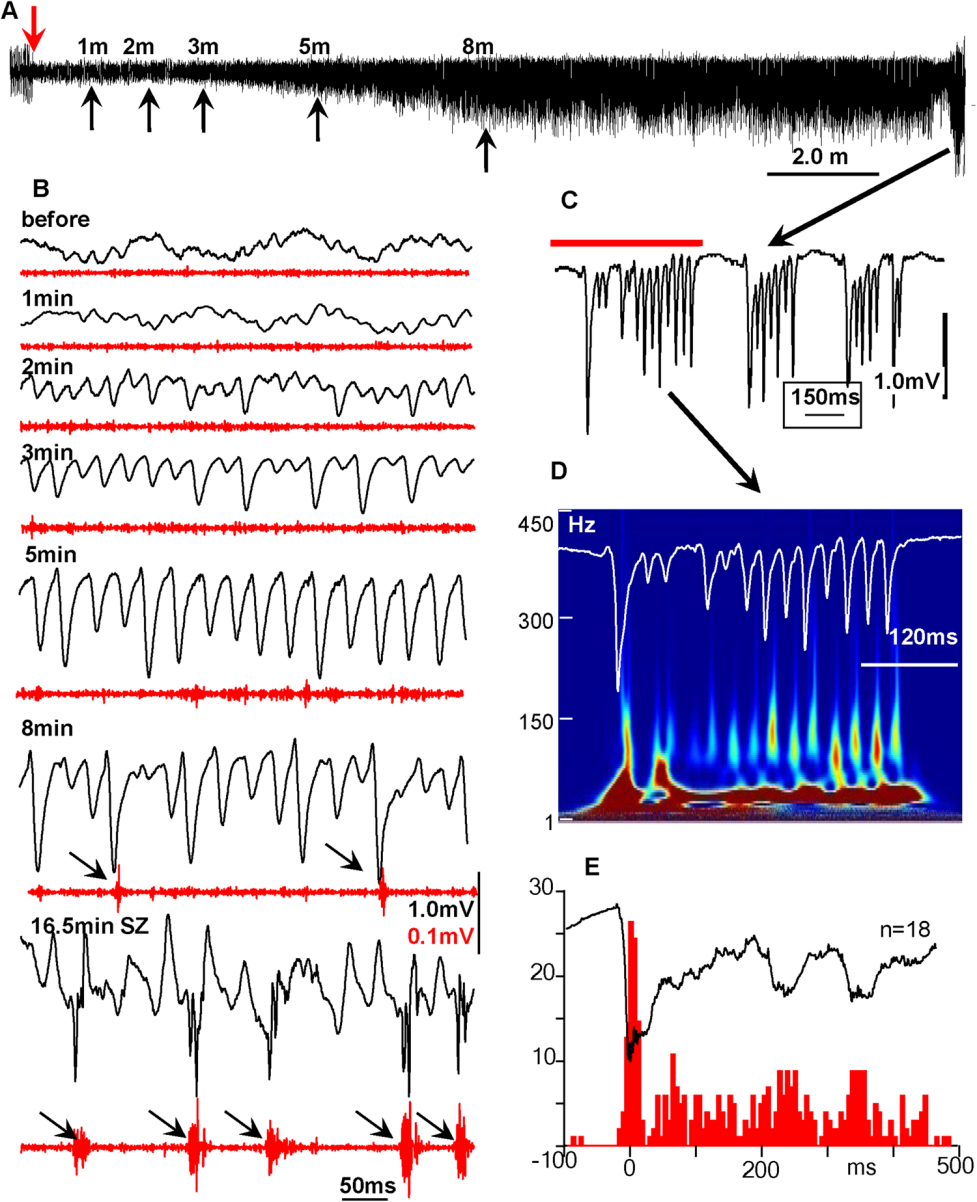
Electrophysiological patterns of seizure activities after KA injection. (**A**) Development of a seizure in the CA3 area of hippocampus in the vicinity of the kainic acid injection. The down-facing red arrow indicates the time of kainic acid injection. The up-facing black arrows indicate periods taken for extension in part (**B**). (**B**) Changes in the pattern of LFPs after KA injection. Black lines are raw data and red are multiunit discharges. (**C**) An example of epileptiform discharges during the seizure in the area of injection. Arrows indicate an increase in synchrony of neuronal discharges. (**D**) Time frequency plot of the activity recorded in the injection area in part (**C**) (red line). (**E**) Peri-event histogram of multiunit discharges during the seizure. Numbers in the y axis indicate number of spikes.

KA was injected when animals were awake, and their baseline electrical activity revealed prominent theta rhythm with superimposed gamma activity in most brain areas. The immediate consequence of KA in the injection area was suppression of electrical activity. In different animals, the period of suppression could last from several seconds to several minutes (Fig. 1A). After this temporal suppression, electrical activity, including theta and gamma bands, recovered (Fig. 1B). Then an increase in amplitude of 20–50 Hz (gamma frequency band) occurred, which continued to increase in amplitude for 5–30 minutes, after which the frequency suddenly dropped to 1–3 Hz activity with a combination of slow waves and fast components in the frequency band of 20 to 200 Hz. This is illustrated in Fig. 1B, where an intermittent increase of gamma events with parallel suppression of theta activity appears during the second minute after KA injection. Initially, an increase in gamma event amplitude was not associated with an increase in synchrony of multiunit discharges (MUD). Later (see Fig. 1B, 8 min example), occasional hypersynchronous MUD occurred. Then, during the behavioral seizure (stage 3)^15^, these gamma events transformed into sharp population spikes with a duration of 5–20 ms (Fig. 1B, [8 min and 16.5 min], C), which are represented in the time frequency plot as peaks in the frequency band of 70–200 Hz (Fig. 1D). These spikes were accompanied by hypersynchronous MUD, illustrated in Fig. 1E.

### Propagation of recurrent epileptiform activity

Total latency descriptive data are presented in Table 1. A total of 478 measurements of latencies from SOZ to seven recording areas (see methods) were quantified in this study. Mean propagation latency for the first seizure was 14.95 seconds, with a relatively large variation from a minimum of 0.5 seconds to a maximum of 85 seconds.

**Table 1:** Total and the factor-related seizure latency descriptive data. Abbreviations: SD – standard deviation; SE – standard error of mean; SN – seizure number; dSOZ – distance from Seizure Onset Zone; Long, Medium and Short– areas of brain located from SOZ correspondingly in 10–12 mm, 7 mm and 3 mm. BS – brain side (contralateral or ipsilateral).

	Number of recoding sites	Mean Latency (s)	SD	SE	Min(s)	Max (s)
Total	478	14.95	14.58	0.67	0.5	85
SN						
Seizure#1	97	11.60	9.77	0.99	1	43
Seizure#2	92	15.01	11.98	1.25	0.5	77
Seizure#3	105	14.59	15.01	1.46	1.0	72
Seizure#4	91	15.37	15.74	1.65	0.5	77
Seizure#5	92	18.44	18.46	1.93	1.0	85
dSOZ						
Long	171	19.43	17.55	1.34	1.0	85
Medium	146	15.40	13.27	1.10	1.0	59
Short	161	9.80	9.93	0.78	0.5	55
BS						
Contralateral	244	19.26	16.72	1.07	1.0	85
Ipsilateral	234	10.46	10.20	0.67	0.5	55

**Figure 2:**
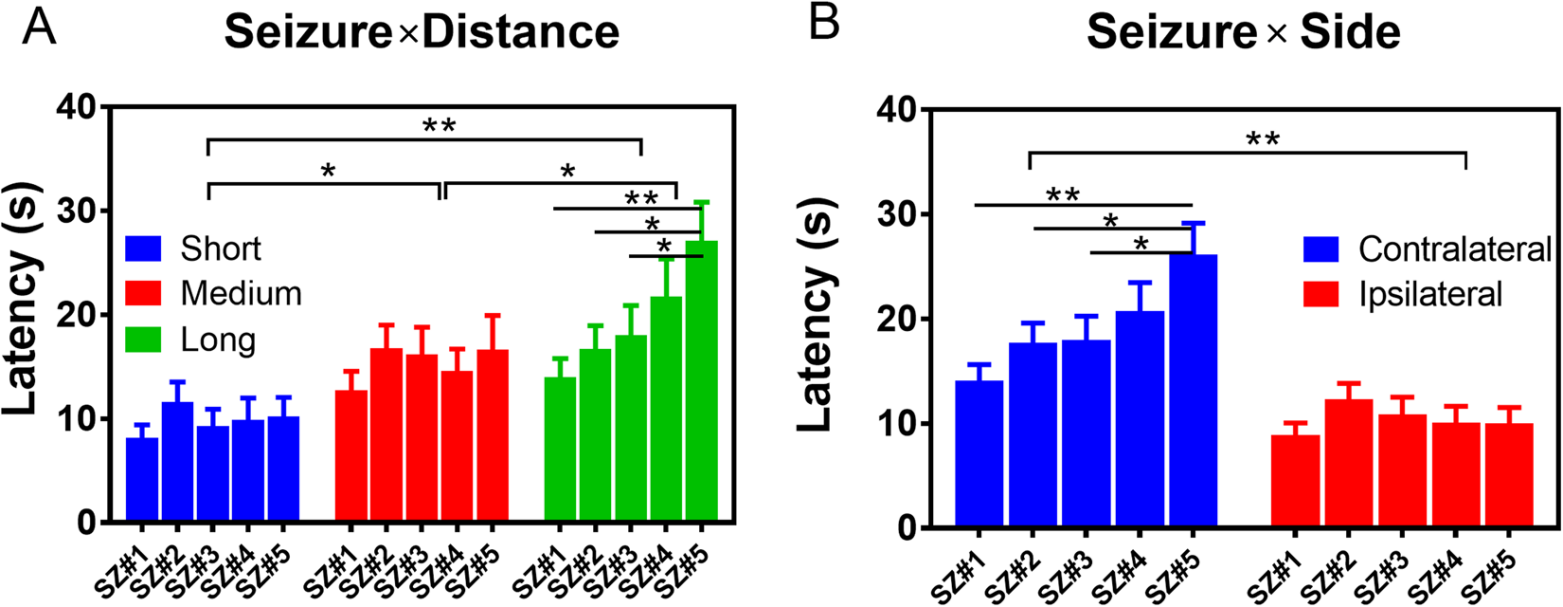
Propagation of seizure activities. (**A**) Mean latency of seizure occurrence in relation to the distance to the recording site from the seizure onset zone for five consecutive seizures in each of 17 rats. (**B**) Mean latency of seizure occurrence in consequent seizures in contralateral and ipsilateral sites.

Specifically, we observed (1) An overall increase in mean latency for each consecutive seizure in long distance case, as indicated in Fig. 2A and Table 1. There is a main effects of seizure number (SN) [F(4, 463) = 2.57, p = 0.03] and distance [F(2, 463) = 19.26, p < 0.001], but not for their interactions (two-way ANOVA, type III). The shortest latencies were observed during the first seizure (short, SZ#1, 11.60 ± 9.77 s), with the longest during the fifth seizure (long, SZ#5, 18.44 ± 18.46 s). Multi-comparisons (Turkey, 95% CI of diff) also revealed a significant difference in mean latency between SZ#1 vs. SZ#5 (p < 0.001, 95%CI = −22.3 to −3.959), SZ#2 vs. SZ#5 (p = 0.02, 95%CI −19.79 to −1.025) and SZ#3 vs. SZ#5 (p = 0.045, 95%CI = −18 to −0.1217) in the long distance group. (2) There is positive association between latencies and the distance between recorded brain areas. The closest brain areas, located at a physical distance of 3 mm [right entorhinal cortex (REC) and right anterior hippocampus (RAH)], showed the shortest latency (9.80 s ± 9.93 s), which gave us an approximate speed of propagation ~0.3 mm/s. The long-distance (10–12 mm) [left posterior hippocampus (LPH), right entorhinal cortex (REC) and left piriform cortex, (LPir)] showed the largest latencies (19.43 s ± 17.55 s), with a mean speed of propagation of ~0.5 mm/s. Multi-comparisons (Turkey, 95% CI of diff) indicated significant differences of mean latencies in Short vs. Medium (p = 0.0014, 95% CI = −9.33 to −1.85), Short vs. Long (p < 0.001, 95% CI = −13.22 to −6.03), as well as Medium vs Long (p = 0.027, 95% CI = −7.71 to −0.34). (3) Differences were also observed in the brain side (BS) group (Fig. 2B). Two-way ANOVA type II tests indicated strong main effects of both seizure number [F(4, 468) = 2.97, p = 0.019] and brain side [F(1, 468) = 49.17, p < 0.001]. Multi-comparisons showed that on the ipsilateral side, mean latencies were not significantly different than on the contralateral side (10.46 s ± 10.20 vs. 19.26 s ± 17.62, t = −1.86, p = 0.06) at a confidence level of 95% CI, but were significant at level of 90% CI. Multi-comparisons (Turkey, 95% CI of diff) also revealed a significant difference of mean latency between SZ#1 vs. SZ#5 (p < 0.001, 95%CI = −19.63 to −4.45), SZ#2 vs. SZ#5 (p = 0.02, 95%CI = −16.2 to −0.70) and SZ#3 vs. SZ#5 (p = 0.02, 95%CI = −15.59 to −0.75) on the contralateral side, but there were no differences between seizures on the ipsilateral site (Fig. 2B).

### Generators of individual epileptiform events

**Figure 3:**
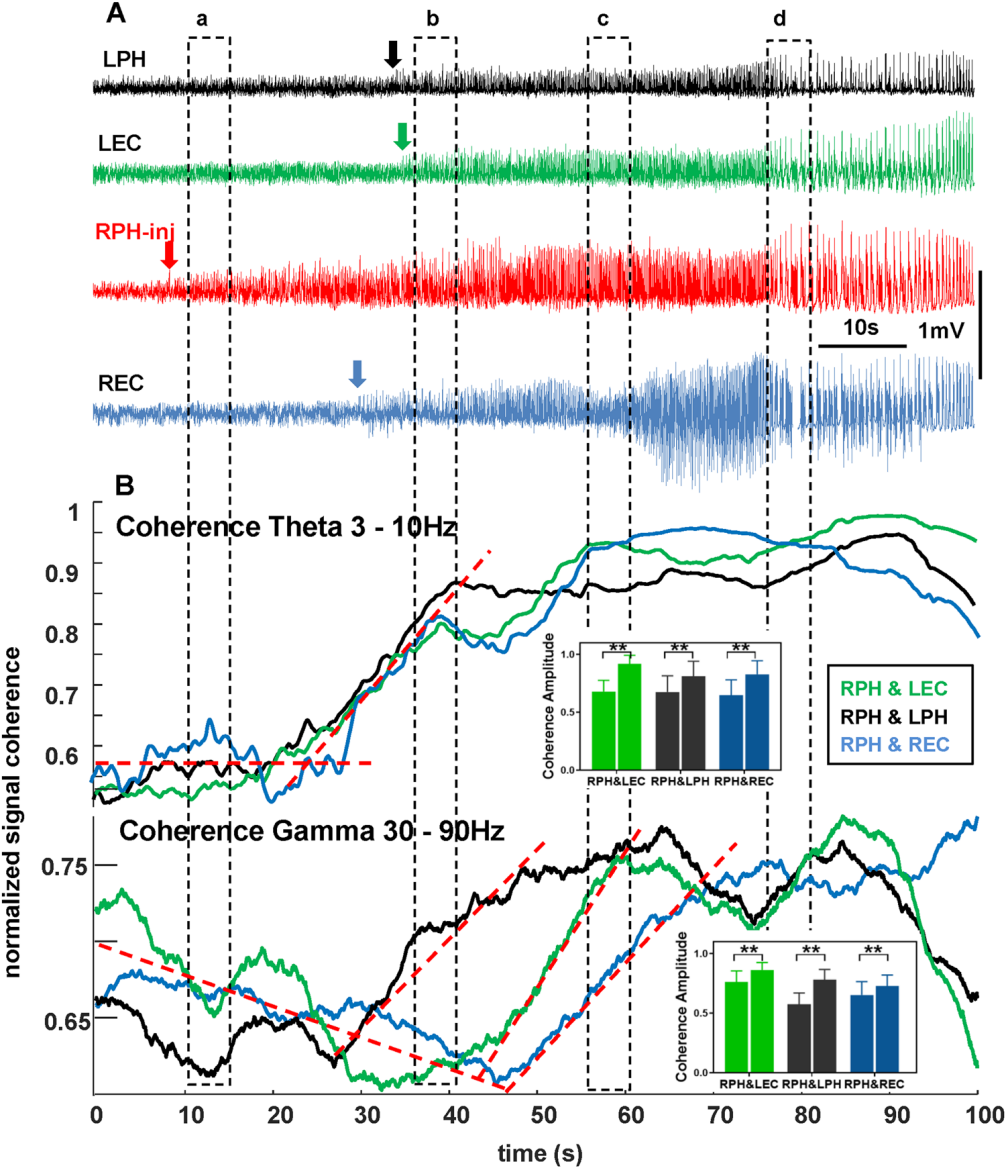
Recruitment of brain areas into the seizure activity after intrahippocampal kainic acid injection. (**A**) Raw data with indication of seizure occurrence in the right posterior hippocampus (RPH-inj – red arrow), in the right entorhinal cortex (REC – blue arrow) as well as in the left posterior hippocampus (LPH – black arrow) and entorhinal cortex (LEC – green arrow). (**B**) Coherence for theta (top) and gamma (bottom) frequency bands during recruitment of different brain areas into the seizure activity. Dashed lines indicate slopes of coherence changes for each frequency band.

We had assumed that epileptiform activity in areas outside the site of injection would be driven by hypersynchronous discharges from the area of injection. Continuous calculation of wavelet magnitude-based coherence provided more comprehensive information about relationships between the SOZ and other brain areas. For all seizures we observed an initial increase in coherence in the theta frequency band after occurrence of seizures at the point of injection, followed by an increase of coherence in the gamma frequency band. Specifically, we observed significant increases of coherence amplitude in RPH&LEC (p < 0.001, 95%CI = −0.11 to −0.087), RPH&LPH (p < 0.001, 95%CI = −0.21 to −0.19) and RPH&REC (p < 0.001, 95%CI = −0.089 to −0.061) in the theta band. Gamma band coherence amplitude also showed a significant increase in these pairs [RPH&LEC (p < 0.001, 95%CI = −0.25 to −0.22), RPH&LPH (p < 0.001, 95%CI = −0.15 to −0.11) and RPH&REC (p < 0.001, 95%CI = −0.19 to −0.16)] (Fig. 3B). A typical example of temporal relationship among epileptiform discharges recorded in four brain areas is shown in Fig. 3. The first sign of epileptiform activity outside the SOZ appeared in the REC after a period of about 20 seconds and 5–6 seconds later in the LPH and LEC. An increase in coherence of electrical activity within the theta frequency band (Fig. 3B) began before the appearance of LFP signs of ictal activity. Coherence in the gamma frequency band showed an initial decrease and later an increase as the seizure progressed (Fig. 3B, bottom section).

**Figure 4:**
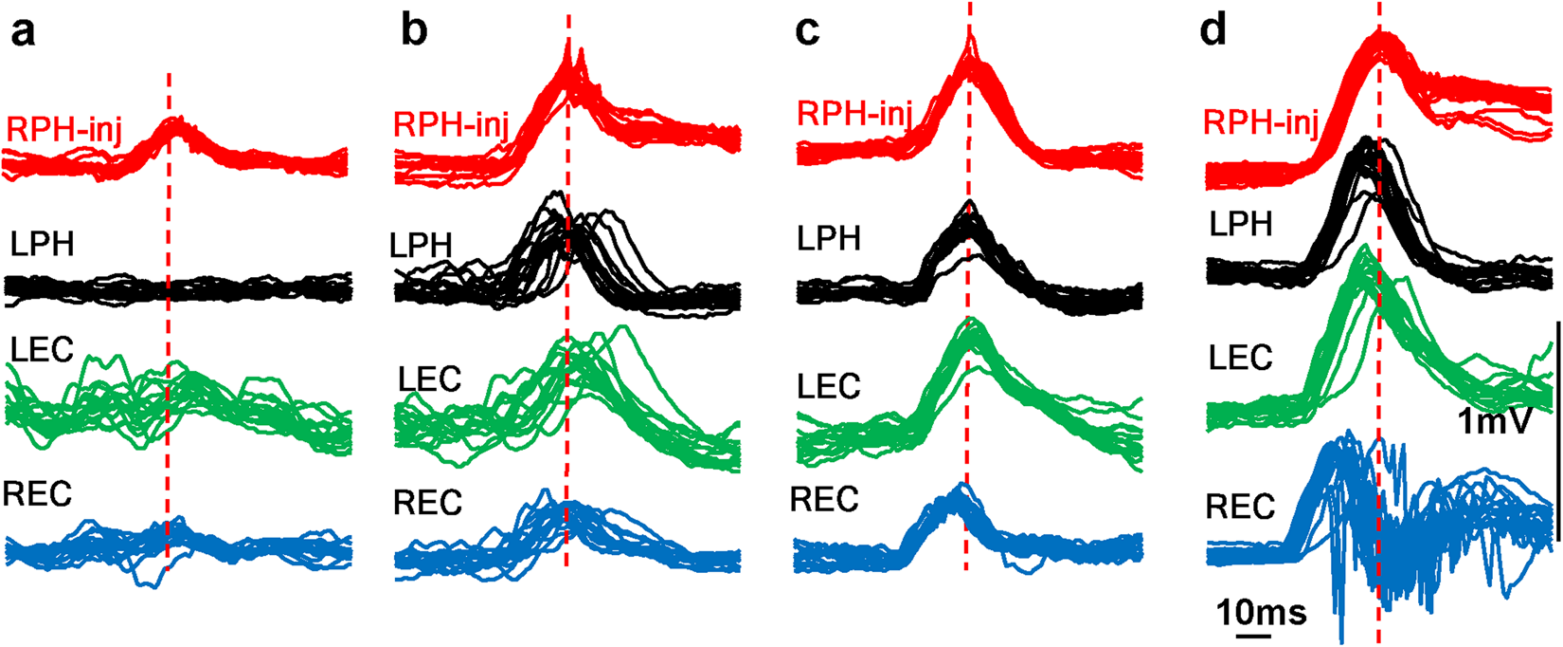
Recruitment of brain areas into the seizure activity after intrahippocampal kainic acid injection. (**a**–**d**) Are time periods during the seizure presented in Fig. 3 (rat #10 in the Table 2. Each column presents superimposition of single seizure events (n = 15) in relation to the peak of the amplitude of seizure events (dashed lines) recorded in the area of injection (red).

**Figure 5:**
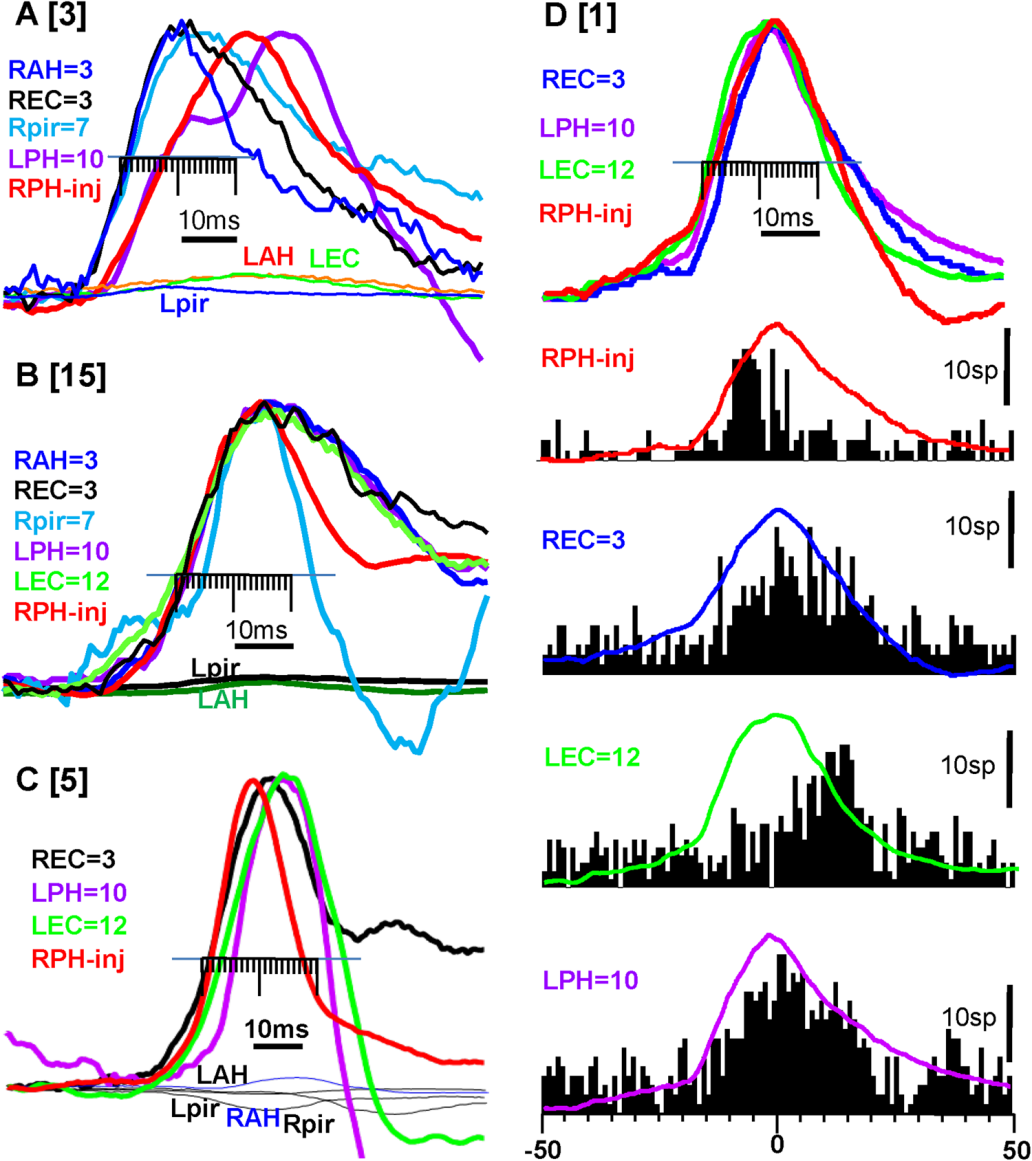
The time difference between epileptiform events recorded in the area of injection (red line) and other brain areas after injection of kainic acid in 3 different rats (**A**–**C**) [numbers in square brackets are identical with the rat number in the Table 2]. The average of the first 10 epileptiform events from the seizure onset normalized by amplitude are presented in each graph. The areas which show epileptiform events are indicated on the left of each graph and color matched to the lines in the graph. The numbers indicate the physical distance between the recorded site and the injection site in mm. The minor division on the scale is 1ms. Shaded lines indicate SD Abbreviations are the same as on the Fig. 2D. Correlation of multiunit discharges and LFP during seizure development after kainic acid injection. The top lines are LFPs for each recorded brain area and below are perievent histograms referenced to the peak of LFP recorded in the injection area.

On the level of micro-intervals, by measuring the time differences *between individual epileptiform events* recorded from the area of seizure onset and other brain areas, we observed the following: Fig. 4 illustrates superimposed LFP events during different times during the development of the seizure presented in Fig. 3. Before the spread of ictal activity (Fig. 4a) there were no visible phase-locked changes in LFPs recorded in other brain areas. At the beginning of the recruitment process there was no relation to, nor high dispersion of, LFP peaks in other brain areas with respect to the peak epileptiform event amplitude recorded in the SOZ (Fig. 4a,b). Later, as the seizure developed, the dispersion of ictal event amplitudes decreased and ictal events in other brain areas located within 3–12 mm distance from the SOZ occurred simultaneously, or even before ictal events recorded in the SOZ (Fig. 4c,d). Additional examples of coherence of ictal activity in three other rats are presented in Fig. 5 and Table 2. Figure 5A illustrates that in RAH, REC, RPir, which are remote from the area of injection, 3–7 mm ictal events occurred 7 ms earlier than at the point of injection, and in parts B,C epileptiform events occurred within 5 ms in all brain areas regardless of their location in the brain. These epileptiform LFPs are associated with an increase in synchronization of multiunit discharges (Fig. 5D) and a peak of synchronization of MUD may occur on ascending as well as on the descending phases of LFPs. Detailed analysis of MUD- LFPs relationship is a subject of another paper.

**Table 2:** The relative latency and standard deviation of epileptiform events in milliseconds in relation to the injection point after kainic acid injection. Numbers in “0” indicate brain areas where seizure events occurred first, and numbers in “1” indicate brain areas where seizure events occurred within 1ms delay. Note: in cases where epileptiform events in other brain areas occurred earlier than in the area of KA injection (rats number 2,3,4,8,10,11), the latency still was calculated as if these events were propagated from the area of injection. n/a indicate those brain areas that were no involved into a current seizure activity.

Rat ID	Areas of recording						
	RPH-injection	RAH 3 mm	REC 3 mm	RPir-7 mm	LAH-7 mm	LPH-10 mm	LEC-12 mm
1	0	n/a	5	n/a	n/a	5	5
2	8	n/a	1	n/a	n/a	n/a	0
3	10	n/a	0	0	n/a	n/a	n/a
4	8	n/a	1	0	n/a	24	n/a
5	0	n/a	3	1	n/a	7	n/a
6	0	n/a	3	n/a	10	8	10
7	0	5		n/a	n/a	1	8
8	3	n/a	0	n/a	33	n/a	n/a
9	0	15	5	n/a	n/a	15	10
10	10	3	0	1	26	10	25
11	10	8	1	8	20	0	n/a
12	0	6	3	4	7	8	9
13	0	3	12	n/a	8	8	15
14	0	1	1	1	n/a	n/a	n/a
15	0	n/a	1	1	1	n/a	1
16	0	n/a	5	10	n/a	1	10
17	0	1	8	n/a	7	n/a	10
Mean ± SD		5.2 ± 4.6	5.0 ± 3.7	5.5 ± 6.3	14.0 ± 11	9.6 ± 6.2	10.1 ± 5.8

Overall, the mean latency of epileptiform events to the areas located ipsilateral to the area of injection varied from 5.0 to 5.5 ms (5.2 ± 3.8 ms), which was significantly shorter (p < 0.001) than the latency of epileptiform events to the contralateral sites (range 9.6–14.1 ms, 11.4 ± 5.9ms). Considering the physical distance between recording sites, we can approximately estimate the speed of propagation of the electrical signal between the area of injection and ipsilateral entorhinal cortex = 0.4 m/s, and between the area of injection and contralateral hippocampus and entorhinal cortex = 0.8–1.0 m/s. However, analysis of individual cases revealed that in ten animals (59%) at least one recording site had only a 1–2ms delay from the epileptiform LFP recorded in the area of injection (see Table 2, numbers in “1”) and in six animals (33%) epileptiform events in areas outside the area of injection occurred 3–10ms earlier than at the site of injection (zeros outside the area of injection).

## Discussion

Some properties of seizures induced by KA injection and their relation to spontaneous seizures where described in our previous publications^16–18^. Here we will focus our discussion on propagation of seizure activity in acute conditions.

There are two findings in this study: (1) After intrahippocampal injection of KA, ictal activity propagates from an acute epileptic focus to other brain areas within a period of several seconds to several tens of seconds, and (2) Once the seizure begins, epileptiform events in target areas are not necessarily driven by epileptiform events from SOZ.

Although the pattern of seizure spread after systemic and intrahippocampal KA injection has been described in numerous publications^19,20^ (see also review Levesque and Avoli, 2013^21^), in the current study we have performed recordings directly from the acute epileptic focus and sampled a high number of target sites during the ictal discharges. Formalized criteria were applied for measurements of latency of propagation of epileptiform activity. The latency between epileptic events in the SOZ and ipsilateral sites was 10.46 sec and to contralateral sites 19.26 sec. The latency of propagation of ictal discharges described in patients with epilepsy is in the same range and varies from 0.6 to 34 seconds^2,9,22^. Considering that a volume of rat brain is ~2 cm^3^ and the size of the human brain is ~1260 cm^3,^ ^23^, we can conclude that the factor regulating propagation of ictal discharges does not necessarily depend on the distance between the SOZ and target brain areas. Moreover, such speed is too slow to explain propagation via simple synaptic or non-synaptic pathways. As indicated in several previous publications, slow propagation of ictal activity is mainly influenced by seizure-suppressing mechanisms in each individual brain area. There are several potential mechanisms opposing seizure propagation: homeostatic, ionic, and metabolic^24,25^, and so far only one of them, feedforward inhibition, has been described in detail for propagation of neocortical seizures^26–31^.

Similar studies are absent for seizures propagating between non-neocortical brain areas; however we can assume that feed-forward inhibition could be one of the mechanisms that control propagation of ictal discharges between different limbic brain areas.

Our study suggests that innate seizure-preventing mechanisms are different for ipsilateral and contralateral connections. The latency of seizures propagated to ipsilateral sites did not change for repeated seizures, while in contralateral target areas it increased for each consecutive seizure. At present, it is not clear whether the strength of feedforward inhibition between the SOZ and contralateral target areas becomes stronger after repeated seizures, or whether other properties of seizure-preventive mechanisms are involved in this process.

The occurrence of a seizure is considered to be involved in the formation of neuronal networks with specific functional connections between brain areas involved in ictal activity^24,32–37^, where recorded structures can be considered as *nodes* and functional connections between them as *edges*. There is controversy regarding how network elements functionally interact during a seizure. According to some data before and during seizure activity, elements of the seizure network become desynchronized^33,38^, while other publications describe an increase in synchronization measured by different methods^36^. One of the reasons for this controversy could be that there are different types of experimental seizures used for analysis, and different frequency bands used for assessing of coherence. In our experiments, involvement of brain areas into ictal activity was associated with an increase in spatial synchronization of electrical activity between SOZ and target areas in the theta frequency band and later in the gamma frequency band. It is logical to assume that coupling of individual ictal events contributes to increased coherence, and these individual ictal events could be driven by epileptiform events from SOZ. It would indicate that epileptiform events from SOZ are connected via morphological pathways to target areas, and we would expect dependence of the latency of epileptiform events from the SOZ to the target areas to be dependent on distance. However, as illustrated in Fig. 3 an increase in coherence between SOZ is different with different brain areas and does not depend on the distance between SOZ and recorded brain area. Moreover, in almost 60% of rats, epileptiform events in target brain areas occurred within only a 1ms delay from epileptiform events recorded in the SOZ, or even before. This may indicate the existence of a common drive for all epileptiform events during some seizures. Such a common drive could be the septum. Indirectly, this indicates an increase in coherence in the theta frequency band at the seizure onset and the septum is a main generator of theta rhythm^39,40^. Some publications have suggested the importance of septum in propagation of hippocampal seizures^41,42^; however, none measured the temporal relationship among individual epileptiform events recorded from multiple brain areas. Direct measurements from septum and other brain areas are necessary to enable us to explore its role in driving epileptiform events during seizures. Another explanation for the near-simultaneous occurrence of ictal events in remote brain areas during a seizure would be the oscillating networks and coupling between epileptiform events in the SOZ and target brain areas due to stochastic resonance mechanism, as described in some publications^43–45^.

## Methods

Adult Sprague-Dawley rats (male, weight 250–300 g) were used in these experiments. All procedures described in this study were approved by the Institutional Animal Care and Use Committee of the David Geffen School of Medicine and experiments were performed in accordance with the relevant guidelines and regulations of NIH. A total of 17 rats were used in these experiments, a total of 1,421 seizures were recorded, and we limited our analysis to 85 seizures, consisting of the first five seizures in each animal.

### Microelectrode implantation

Animals were anesthetized with isoflurane and fixed into a stereotaxic frame. A guide cannula (200 µm OD) was implanted into the CA3 region of the right posterior hippocampus (RPH), coordinates according to^46^: anterior–posterior (AP), −5.0; medial–lateral (ML), 5.0; dorsal–ventral (DV), 5.3. The outer part of the cannula’s trunk was covered by varnish while the tip was exposed to record electrical activity at the site of injection and the magnet wire was soldered to the upper part of the cannula. Fixed recording microelectrodes consisting of tungsten wire (50 um outer diameter) were implanted into the ipsilateral anterior hippocampus (RAH, AP, −3.5; ML, 2.0; DV, 4.5), ipsilateral entorhinal cortex (REC, AP, −7.0; ML, 5.9; DV, 7.0), ipsilateral piriform cortex (RPir, AP, 1.0; ML, 4.5; DV, 7.5), and symmetrical contralateral sites (LAH, LPH, LEC and LPir). These areas were chosen because they are part of hippocampo-enthorhinal circuitry. Piriform cortex was selected for this study as a part of area of tempestas, which has a low threshold for seizure activity^47^.

### Data acquisition

Experiments began one week after surgery. Recordings of electrical activity were performed in freely-moving conditions. Data were recorded wide-band 0.1 Hz to 3.0 kHz and sampled at 10 kHz per channel (16 channels) using Run Technologies DataPac 2K2 software.

### Injection protocol

After one of baseline recordings, KA was injected into posterior CA3 with a 10 µl Hamilton syringe that extended to a needle that fit into the injection cannula, which was 0.5 mm longer than the implanted guide cannula. KA was injected at a concentration of 8.6 mM in a volume of 0.2 µl and a flow rate of 0.1 µl/10 s.

### Histological procedures

At the end of the electrophysiological experiments, rats were deeply anesthetized and perfused with 2.5% paraformaldehyde. Brains were removed and placed in 2.5% paraformaldehyde for at least 48 h before histological sectioning and Nissl staining to verify electrode placements. On the horizontal histological sections, the electrode track was traced downward until it disappeared from the section, and the last section in which each electrode’s track was visible was considered the site of recording. The location of recording sites from all 17 rats is presented in Supplement Fig. 1.

### Calculation of the volume of kainic acid spread

In three rats, 6 injections of methylene blue (20 nmol^48^), were injected into the posterior hippocampus with the same speed and the same location as for the kainic acid injection (see Methods). Rats were euthanized one hour after injection and perfused with paraformaldehyde. After 24 hours, brains were sliced with a vibratome (Leica, model 1000-S) on 100 µm sections, and were reviewed under a surgical microscope. Tracks from the injection cannulae were identified and the diameters *d*_*i*_, *i* = 1, …, *n* (*n* = total number of slices) of the visible blue spots were measured in each slice. The volume was calculated by the formula: $V=\sum _{1}^{n}\pi \times ({d}_{i}/2)^{2}$. The injected volume of KA was 0.2 µl. In these modeling experiments with injection of the same volume of methylene blue (n = 6) we found that the dye spread a distance of 0.3–0.6 mm from the point of injection, mean 0.48 ± 0.12 (SD), which is within the injected CA3 area. (see example in Supplement Fig. 2).

### Data analysis

After histological verification of recording sites, the physical distance between the area of injection and each recording area was measured using a stereotaxic atlas^46^. On the basis of these measurements, the mean distance between the area of injection and the recording brain areas was the following: RAH = 3; REC = 3 mm; rPir = 7 mm; LAH = 7 mm; LPH = 10 mm; LPir = 11 mm; LEC = 12 mm. Data analysis focused on two questions: (1) propagation of ictal activity from the area of injection, which we considered as the seizure onset zone (SOZ), estimated by measurement of the latency to seizure occurrence in other recorded brain areas; and (2) generation of ictal discharges during ictal activity, which was estimated by calculating the temporal relationship among individual epileptiform events recorded from the SOZ and other brain areas.

Analysis was performed using DataPac software. High-pass filtering (1 Hz, butterworth, rolloff 3, with zero lag compensation) was applied to the raw data to remove the slow component of electrical activity. For detection of multiunit activity raw data were filtered by a high-pass filter (300 Hz, butterworth, rolloff 3, with zero lag compensation) and a threshold of 2 SD was set to detect unit discharges. The seizure onsets at the point of injection and at other brain areas were identified at the time when the amplitude of electrical activity crossed the threshold of 1-SD of the mean amplitudes for a period longer than 10 seconds. The propagation latencies in areas outside the SOZ were computed as the time difference between the beginning of seizures in the SOZ and the beginning of ictal discharges in recorded brain areas. Means, standard deviations, and ranges for seizure latency data were computed. Analyses of variance tests (ANOVA) were performed to investigate possible relationships of seizure onset latencies to seizure numbers (SN), distances from seizure-onset-zone (dSOZ), and location of brain areas in ipsilateral or contralateral brain sites (BS). Specifically, there were five levels of SN, sorted by the occurrence of seizures, namely, seizure #1 - #5; three groups of dSOZ: short (3 mm from the SOZ), medium (7 mm from the SOZ) and long (10–12 mm from the SOZ). In addition, BS was defined as either “ipsilateral” or “contralateral” to the (dSOZ). We considered the dSOZ and the BS as two unrelated factors because both are categorical variables of description of locations. Therefore, two separate statistical analyses were performed: (1) to test the main effects of SN and dSOZ and their interactions (SN $\times$ dSOZ) in seizure latencies, and (2) to examine the effects of SN and BS and their interaction (SN $\times$ BS) in the variance of seizure latency.

Analysis of generators of ictal discharges was performed by calculation of the coherence of electrical activity between the SOZ and other brain areas, as well as by the measurement of the temporal relationships between *individual epileptiform events* recorded in the SOZ and other brain areas. The peaks of epileptiform events in the SOZ were detected using DataPac software. Then event-triggered averages of signals recorded in other channels were determined. Amplitudes of averaged events were normalized and time differences between SOZ and other brain areas were calculated based on a point at half-amplitude of each epileptiform event (see Fig. 5 for illustration).

## Electronic supplementary material

Supplementary Information
