# Supplementary material for: Seizure development in the acute intrahippocampal epileptic focus
Source: Sci Rep. 2018 Jan 23;8:1423. doi: 10.1038/s41598-018-19675-6 (PMC5780458; doi:10.1038/s41598-018-19675-6)
Supplement: Supplementary file 1 — Supplementary Information [file 41598_2018_19675_MOESM1_ESM.pdf]

Supplementary information

Title: Seizure development in the acute intrahippocampal epileptic focus

Authors: Lin Li[1], Kseniia Kriukova[1,5], Jerome Engel Jr [1,2,3,4 *], Anatol Bragin[1,4 *],

Affiliations: [1]Department of Neurology, [2]Department of Neurobiology, [3]Department of Psychiatry and Biobehavioral Sciences, [4]Brain Research Institute, David Geffen School of Medicine at University of California Los Angeles, 710 Westwood Plaza, Los Angeles, CA, USA, 90095; [5]I.M. Sechenov First Moscow State Medical University, Moscow, Russia

Corresponding author:

Anatol Bragin, email: abragin@mednet.ucla.edu. Department of Neurology, David Geffen School of Medicine at University of California Los Angeles, 710 Westwood Plaza, Los Angeles, CA, USA, 90095 (phone:818-414-4096),

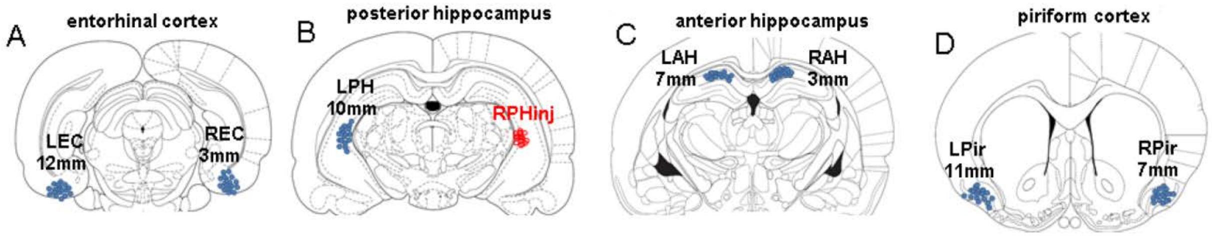

Supplement Figure 1. Distribution of electrode location based on histological evaluation of 17 rats. A, entorhinal cortex; B, posterior hippocampus, red dots are location of kainic acid injections; C, anterior hippocampus; D, piriform cortex. Letters inside each section indicate abbreviation of each recording site and numbers indicate physical distance from the pint of kainic acid injection.

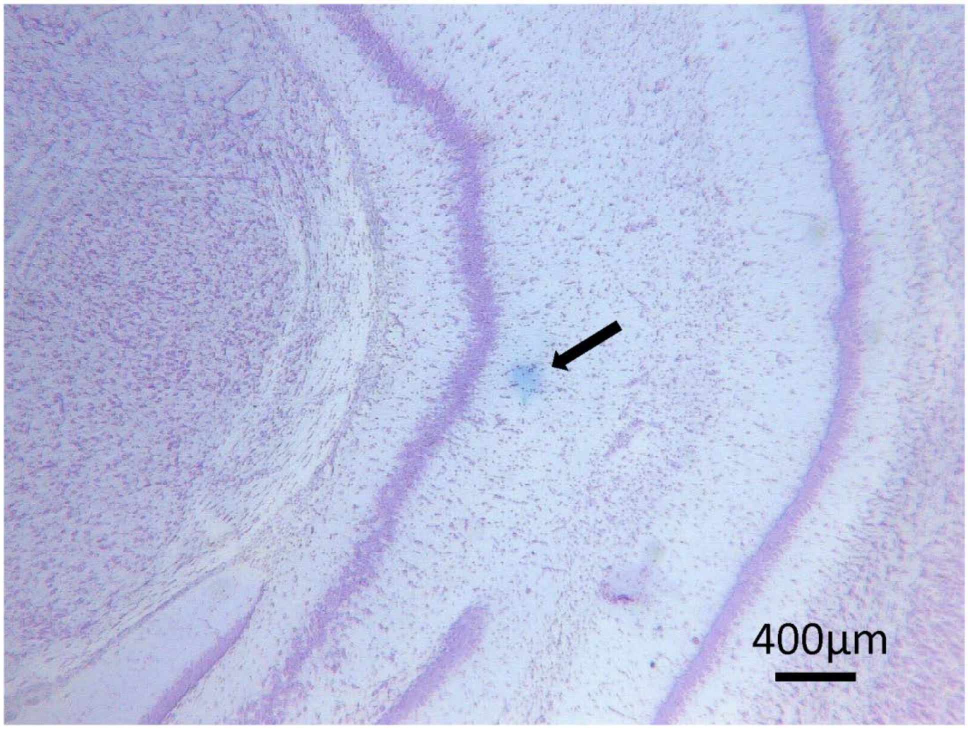

Supplement Figure 2. An example on methylene blue injection (black arrow) in the rat posterior hippocampus.
